# A Multi-Modal Egocentric Activity Recognition Approach towards Video Domain Generalization

**DOI:** 10.3390/s24082491

**Published:** 2024-04-12

**Authors:** Antonios Papadakis, Evaggelos Spyrou

**Affiliations:** 1 Department of Informatics and Telecommunications, National Kapodistrian University of Athens, 15772 Athens, Greece; antonispapd@gmail.com; 2 Department of Informatics and Telecommunications, University of Thessaly, 35100 Lamia, Greece

**Keywords:** visual transformers, egocentric vision, multi-modal activity recognition, domain generalization, domain adaptation, egocentric activity recognition

## Abstract

Egocentric activity recognition is a prominent computer vision task that is based on the use of wearable cameras. Since egocentric videos are captured through the perspective of the person wearing the camera, her/his body motions severely complicate the video content, imposing several challenges. In this work we propose a novel approach for domain-generalized egocentric human activity recognition. Typical approaches use a large amount of training data, aiming to cover all possible variants of each action. Moreover, several recent approaches have attempted to handle discrepancies between domains with a variety of costly and mostly unsupervised domain adaptation methods. In our approach we show that through simple manipulation of available source domain data and with minor involvement from the target domain, we are able to produce robust models, able to adequately predict human activity in egocentric video sequences. To this end, we introduce a novel three-stream deep neural network architecture combining elements of vision transformers and residual neural networks which are trained using multi-modal data. We evaluate the proposed approach using a challenging, egocentric video dataset and demonstrate its superiority over recent, state-of-the-art research works.

## 1. Introduction

Humans have been fascinated with capturing moments in their lives and preserving them in time since the pre-historic period. Cave murals, paintings, monuments and drawings, ranging all the way from battling to performing daily tasks, such as preparing a meal and eating, stand still as evidence of daily human activities throughout history. The invention of analog and more recently of digital photography offered the means to obtain, store, review and process a large volume of human-centered visual data. These advancements ultimately gave birth to computer vision and tasks such as face/object recognition, emotion recognition and human activity recognition. These applications have benefited tremendously from the recent advances in the fields of artificial intelligence and hardware accelerators, bringing us firstly into the era of machine learning and more recently into the one of deep learning. The latter is characterized by the lack of need for the extraction of handcrafted feature representations, replacing them with features that are “learned” from deep neural networks.

One of the fields that has significantly benefited from the aforementioned advances is the one of Human Activity Recognition (HAR) [1,2,3]. HAR approaches may be applied in several tasks, such as video surveillance [4], health/elderly care [5], human–computer interactions and/or automation [6,7], sports analysis/training [8], behavior analysis [9] etc. HAR methodologies may be categorized into two main categories, i.e., sensor- and vision-based [10]. The former uses analytics on raw sensor measurements, while the latter is based on visual data.

Over the last few years several research efforts on vision-based HAR have turned to egocentric/first-person activity recognition, which focuses on videos typically captured using wearable cameras. Thus, egocentric videos are captured through the perspective of the camera wearer, resulting in footage characterized by significant, non-linear and unpredictable movements of the head and body, which in turn are responsible for a lack of a global context [11]. With the advent of wearable cameras such as GoPro and other similar products, the amount of egocentric data has significantly increased. Recently, large and challenging datasets comprising egocentric videos of human actions have provided new opportunities in developing robust recognition models. Notable examples of such datasets include the original Epic-Kitchens-55 dataset [12] as well as its extended version, i.e., Epic-Kitchens-100 [13], comprising 55 and 100 h of daily activities in the kitchen using head-mounted cameras, the Ego4D dataset [14], which includes 3670 h (i.e., approx. 5 months) of daily-life activity videos spanning hundreds of scenarios and a novel egocentric object tracking dataset, namely the TREK-150 [15], which is composed of 150 densely annotated video sequences.

In this paper we propose a robust egocentric model that aims to provide an equivalent performance both in the case of (a) evaluation with a dataset that belongs to a feature space similar/adjacent to one of the training sets and (b) when key dissimilarities are present between the training and evaluation sets. Specifically, our efforts have focused on the creation of a model which is able to showcase consistent performance, being independent of factors such as subject, time, location and use case when deployed in a certain scenario setting. For example, let us consider a typical health/elderly care assistive living scenario, wherein such a model may successfully monitor activities performed by the subject in, e.g., any room of any nursing home at any time of day, while being trained only with data coming out of a single room of a single nursing home at a specific time frame. Therefore, we aim to prove that the proposed methodology will be able to produce transferable predictive models used in egocentric HAR scenarios.

Specifically, the herein proposed approach introduces the following novelties towards producing a domain agnostic egocentric HAR model:

- We implement a novel three-stream deep neural network architecture, combining elements of visual transformers [16] and residual neural networks [17], able to be trained with multi-modal data, which in our case comprise raw RGB videos, optical flow and audio data;
- We incorporate the audio modality in the process of egocentric recognition by using the spectrogram transformations of audio data and we demonstrate that this could significantly improve recognition performance;
- We propose a novel, target domain-flavored data augmentation process which aids in the domain generalization process.

The rest of this paper is organized as follows: In Section 2 we present related work in the areas of supervised activity recognition, domain adaptation and egocentric and multi-modal activity recognition. Then, in Section 3 we present the proposed methodology for egocentric activity recognition. Experiments and results are presented in Section 4. Finally, conclusions are drawn in Section 5, wherein plans for further extensions of the herein presented work are discussed.

## 2. Related Work

### 2.1. Supervised Activity Recognition

Supervised activity recognition (SAR) involves the use of both traditional machine learning (ML) approaches and also of modern deep learning (DL) approaches, with the goal of recognizing human actions, given appropriate data. The latter should be manually annotated into a predefined number of classes. A typical SAR pipeline typically involves a model trained on features of various modalities, extracted from these data. Datasets that are appropriate for SAR usually involve several subjects (“actors”) performing scripted actions in a static setting/background, e.g., as in the cases of PKU-MMD [18] and NTU-RGB+D [19] multi-modal datasets for 3D HAR, which provide recordings of human actions from three different viewpoints in a static studio environment. On the other hand, the Kinetics 700 dataset [20] is a huge dataset comprising clips that have been collected from YouTube and take place in a plethora of heterogeneous environments and setups.

Annotated data are usually pre-processed with several cleaning methodologies prior to being used as input for an ML algorithm. This step may include, e.g., treating actions as signals and then using signal processing techniques to transform them into images [21,22], utilizing low-resolution RGB frames or cropping the central area of the frames [23] or even considering short- and long-term dependencies based on depth [24]. Then, ML/DL algorithms are applied to those data for action recognition. Early classification approaches were based on traditional ML algorithms, such as support vector machines [25] or decision trees [26]. More recent DL approaches are typically based mainly on convolutional neural networks [27]. Pham et al. [28] used residual networks (ResNets), while Tu et al. [29] used a two-stream CNN to encode appearance, motion and the captured tubes of human-related regions. Hybrid approaches combine ML/DL algorithms, either by proposing “mixed” architectures [30] or upon applying successively both types of algorithms [31]. Recently, modern attention-based models such as visual transformers have attracted the interest of the research community, e.g., as in the work of Mazzia et al. [32], who introduced an action transformer for short-time HAR from 2D pose information, or in the work of Plizzari et al. [33], where a spatial-temporal transformer network is used to model dependencies between body joint data provided in skeletal representations of human actors in video data.

### 2.2. Domain Adaptation

However, when working with HAR, a critical problem is the following: what if a given model has been trained for activity recognition in a dynamically changing environment, or, alternatively, what if several environmental parameters such as time of day, video lighting, furniture set up, age and appearance of human subjects, camera resolution etc. change in a dynamic manner? These cases require robust HAR models trained on features extracted in a “non-environment-specific” way. A popular option to solve this problem is the use of *domain adaptation* techniques [34].

The goal of domain adaptation algorithms is to create ML models that will demonstrate robust performance when applied to a different domain [35]. By “domain” we refer to the feature space that describes the problem at hand [36]. Specifically, when training a model for a given problem, the specific domain (“source” domain) would be defined from the set of extracted/learned features from the available dataset. It should be evident that the data distribution resulting from feature selection will influence the performance of the model and will introduce bias in the training process. Since datasets are rarely, if ever, adequately large, if the data distribution of the testing set (“target” domain) differs from the one of the training set, a “domain shift” is present. If this is not tackled, it is prone to lead to the poor performance of the ML model. Domain adaptation techniques aim to mitigate the negative effects of domain shift.

Domain adaptation approaches fall under two main categories, namely, unsupervised and supervised. In supervised domain adaptation, a labeled set of data from the target domain is available and may be used to guide the adaptation process. An example of supervised domain adaptation is the work of Goodman et al. [37] whose approach was based on the transfer of the gradient history of the pre-training phase to the fine-tuning phase, while also trying to improve generalization by optimal parameterization during the pre-training phase. Liu et al. [38] exploited generative adversarial training with cycle consistency constraints, enabling a cross-domain style transformation. On the other hand, Ganin and Lempitsky [39] showcased an unsupervised domain adaptation scheme, assuming lack of access to any labeled data from the target domain. In between there exist semi-supervised approaches such as the one of Yan and Lin [40], where a model is trained using a few labeled and significantly more unlabeled target domain data.

Usually, the aim of domain adaptation is to align the target data to the labeled source data with feature space mapping methods. Such methods include the following:

- *Domain adaptation through feature alignment*: in this approach, the features of the source and target domains are aligned to reduce the distribution gap. This can be achieved through techniques such as (a) maximum mean discrepancy, as in, e.g., the work of Long et al. [41]; (b) correlation alignment, as in, e.g., the work of Sunet and Saenko [42]; and (c) adversarial training, as in, e.g., the work of Pei et al. [43]. The last technique is regarded as the prevalent method for domain adaptation through feature alignment.
- *Instance re-weighting*: this technique involves re-weighting training data to reduce the difference between the distributions of the source and target domains and may be achieved through approaches such as importance weighting and covariate shift. An example of the first approach is the work of Adel et al. [44] who used a covariate shift domain adaptation algorithm, considering that both source and target domain labeling functions are identical with a certain probability. Moreover, an example of the second approach is the work of Li et al. [45], where predictions of the training classifier are re-weighted based on their distance to the domain separator.
- *Domain adaptation through data augmentation*: in this approach synthetic data from the target domain are generated and added to the training data to improve model performance, e.g., as in the work of Sarwar and Murdock [46].
- *Transfer learning*: this approach involves the transfer of knowledge from a pre-trained model on a related task to the target domain and is typically countered with several strategies. For example, instead of training a model from scratch, transfer learning leverages the knowledge gained from a source task to improve performance on a target task [47].

### 2.3. Egocentric Activity Recognition

As it should be now evident, egocentric videos refer to media content captured from a human’s point of view. This is typically achieved by using a camera that has been placed as close to the eyes as possible, that is, either on top of the actor’s head or in front of the actor’s eyes, i.e., as video recording glasses. Egocentric view videos are also referred to as “first person perspective” (FPP) or “subjective camera” videos. Currently, with the widespread availability and popularity of wearable cameras such as the GoPro, egocentric videos are becoming increasingly popular and are used for a variety of applications, given their immersive and unique viewpoint and experience, while they are also extremely useful from an engineering standpoint [11]. Typical approaches propose the use of multi-stream deep architectures [29,48] and aim to learn deep, transferable features from multiple modalities [49,50] or modify the statistics of the layers of the deep architectures to remove bias [51]. Several adaptation approaches are often used to handle the divergence between domains [52].

### 2.4. Multi-Modal Activity Recognition

In Section 2.1, we briefly mentioned several common methods that are applied in SAR. This variety of methods is partly promoted by the availability and the diversity of HAR datasets, i.e., apart from RGB video sequences, they also offer a variety of other data modalities, which require different pre-processing, cleaning and/or training approaches. Typical visual modalities that are encountered in this context are depth maps, i.e., the distance of each pixel to the camera viewpoint and skeletal information, i.e., 3D coordinates of a set of skeleton joints per video frame. Moreover, in video datasets, often the audio modality is present, albeit seldom used by the majority of recognition approaches. These modalities when used simultaneously within a given HAR methodology are able to provide complementary insights into the regular RGB data that mainly capture the color and texture image properties of both the actor and the scene.

The spatio-temporal information provided by the aforementioned modalities paves the way for treating human actions as a set of signals [21,22], one/more per data modality. Terreran et al. [53] proposed a multi-modal approach using RGB and depth data given as input to graph convolutional networks. Zhu et al. [54] introduced a bimodal recognition model based on a video and an audio transformer. Similarly, in the work of Ijaz et al. [55] a transformer approach using accelerations and positions of skeletal joints was presented.

## 3. Methodology

In this section we introduce our egocentric view activity recognition machine learning pipeline. Specifically, we present a new Multi-modal Domain Generalization model for Activity Recognition (MDGAR) from videos, (https://github.com/thevisionlab-uth/MDGEAR) (accessed on 12 February 2024) which could also be considered a semi-supervised domain adaptation approach. We follow a training/testing protocol such as the one proposed by Munro and Damen [48], where the recognition model is evaluated using a subset of the largest action classes. To train our network we use one-hot encoding labels. For example, given a set of labels $\left[\mathrm{put},\;\mathrm{give},\;\mathrm{fry}\right]$ and a single action give, the corresponding one-hot encoded label for it would be $\left[0,\;1,\;0\right]$.

### 3.1. RGB Modality

**Figure 1:**
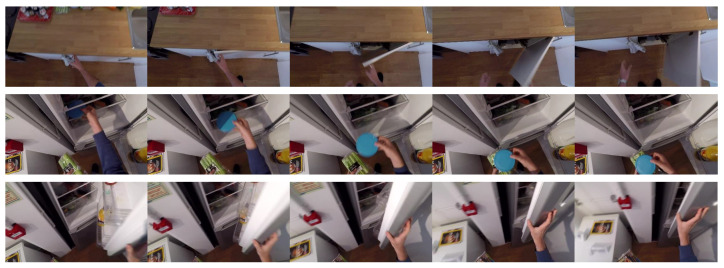
Examples of RGB frames extracted from video sequences of the Epic-Kitchens-55 dataset. From top to bottom, actions (verbs) are “open”, “take” and “close”.

This modality concerns typical RGB videos that are captured using typical wearable cameras. Specifically, in our case, raw videos are processed so as to extract video frames without any additional manipulation. In Figure 1 we illustrate examples of actions “open”, “take” and “close”.

### 3.2. Optical Flow Modality

**Figure 2:**
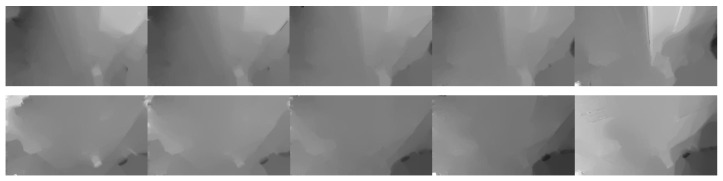
Examples of the optical flow field computed from a video sequence of the Epic-Kitchens-55 dataset depicting action (verb) “take”. Top: *u*-component; bottom: *v*-component.

Optical flow aims to quantify the motion between a series of images that in most cases differ only by a small step in time. Specifically, optical flow calculates a velocity for each point within the image and consequently provides an estimation of the points’ position in the image that follows the aforementioned time step. In our case the sequence of images comprises consecutive frames that constitute the action video, thus the time step is equal to the difference from one frame to the next within the sequence. For the optical flow modality we utilize the already available data of the Epic-Kitchens-55 dataset, which provides in image form both components (u, v) of the optical flow vector. In Figure 2 we illustrate examples of the optical flow field.

### 3.3. Audio Modality

**Figure 3:**
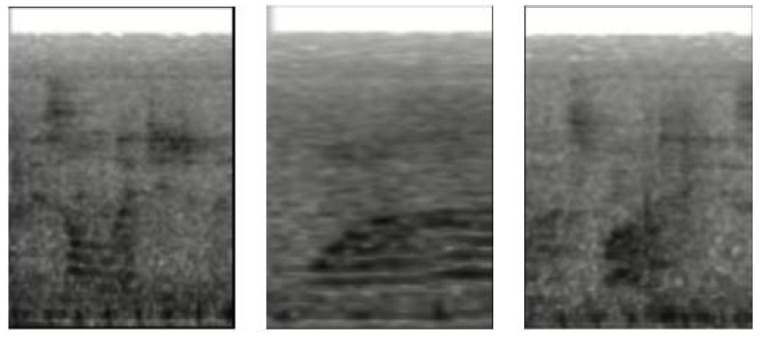
Examples of spectrograms produced from the Epic-Kitchens-55 dataset. From left to right actions (verbs) are “open”, “take” and “close”.

This modality refers to the audio data that are available in the dataset’s videos. During the performance of these actions, audio may originate from activities such as opening/closing cupboards, washing pans, cutting vegetables or pouring liquids in a pot. Note that the Epic-Kitchens dataset does not provide extracted audio recordings of videos. Therefore, we used the available raw video recordings and extracted audio data using the moviepy (https://pypi.org/project/moviepy/ (accessed on 20 February 2024) ) Python library, version 1.0.3. These data were then transformed using the 2D Discrete Fourier Transform (DFT) to spectrograms. We herein remind that a spectrogram corresponds to a 2D image representing signal frequencies vs. time. In Figure 3 we illustrate several spectrogram examples, resulting from processing of the aforementioned audio data. To create spectrograms we used the librosa Python library [56], setting hop length, number of bins and number of time steps equal to 512, 128 and 384, respectively. Note that in these spectrograms, frequencies are displayed in grayscale, i.e., the darker the color gets, the more energy is present in the signal. Ultimately, spectrograms were resized to 128×90 so as to be used as input in our ML models.

### 3.4. Sampling and Scaling

In both visual modalities we applied a pre-processing step to perform sampling of the action sequence within a given temporal window, which in our case was equal to 16 frames. This means that for a given action, several such windows were used for sampling, so as to create a smaller video representation. Let $a_{s}$ and $a_{e}$ denote the starting and ending frames of a given action, and $f_{s}$ and $f_{e}$ the starting and ending frames of a sampled action, respectively. Moreover, let TW denote the number of frames of the temporal window used for sampling and *d* the number of the windows that will be used for sampling a given action. Then, $f_{s}$ and $f_{e}$ are given by:

(1) $$f_{s}=a_{s}+\left(d\cdot \frac{TW}{2}\right)\;,$$

and

(2) $$f_{e}=a_{e}-\left(d\cdot \frac{TW}{2}\right)\;.$$

For each of the temporal windows, its median frame was selected and then TW/2 frames were sampled to its left and right. Note that the aforementioned process was the same for each component of the optical flow vector, with the addition that both flow components were stacked into a single 2D image.

Following this sampling process, the next step was to scale all sampled actions to a uniform size. In cases where the number of sampled frames was smaller to a pre-defined max sequence size $N_{max}$, we used zero padding. On the contrary, in cases where the number of sampled frames exceeded $N_{max}$, an averaging operation was applied to provide a down-scaled image. Specifically, we split the total number of frames *N* to equal parts according to the $N_{max}$, and we calculated a new “average frame” $f_{i}$ between the *i*-th and the (i+s)-frame for each of the parts by iterating through them as follows:

(3) $$f_{i}=\sum_{i=1}^{N}\frac{\sum_{j=1}^{i+s}f_{j}}{s}\;,$$

where $s=N/N_{max}$.

### 3.5. Data Augmentation

During the data augmentation step, available images from all modalities were transformed to produce slightly altered copies of themselves and were used to augment the dataset so as to smooth differences in size between action classes. For the RGB and optical flow modalities transformations used to augment the dataset, minor manipulations of the image zoom level, contrast and rotation were included. Specifically, we used random amounts of zooming, in the range of [−30%,+30%], random amounts of contrast adjustment with a contrast factor in the range of [0,1] and random rotation angles in the range of [−40%·2π,+40%·2π].

For the audio modality a combination of frequency and time random masking was deployed, similar to the work of Kim et al. [57], who applied such masks to effectively preserve the spectral correlation of each audio sample.

However, we should herein note that since our goal was to solve a domain generalization problem, we modified the classic data augmentation process as follows: instead of balancing a given class vs. classes belonging to the same domain, i.e., performed by the same actor, we performed an extra domain balancing process. Specifically, we balanced a class belonging to the source domain to the same class of the target domain. We remind the reader that as the source domain we considered the set of actions performed by the actor that was used for training our model and as target domain we considered the actions performed by a different actor in a different setting and used for testing. Although no other information rather the target domain’s size was used, we considered that this makes the proposed model a semi-supervised one, since information from the target domain was utilized to improve overall performance.

### 3.6. Machine Learning Model

The next step of the proposed pipeline was the ML model which was used for the recognition of actions. To this end, we created a hybrid approach combining convolutional neural networks with vision transformers, which will be presented in the following subsections.

#### 3.6.1. Inflated 3D Convolutional Architecture

In brief, RGB and optical flow modalities were used to train an inflated 3D convolutional architecture (I3D) [58], pre-trained on ImageNet [59] and Kinetics [20] datasets. The I3D model was designed to process videos on a frame-by-frame basis, i.e., 2D frames are given as input in a 3D format, with time being the third dimension. Convolutional layers with stride 2 were included, followed by a max-pooling layer and numerous inception modules. The latter are CNNs with a single max-pooling layer; concatenation is their main task. This model is called “inflated” due to the existence of many of those inception modules. The final layers of the model are an average pooling layer and a 1×1×1 CNN, used for predictions.

#### 3.6.2. Vision Transformer (ViT)

Upon feature extraction from RGB and optical flow, using the I3D model, features were propagated to a vision transformer. This part of the ML model was based on three blocks of CNN augmented transformers. Features extracted from the I3D firstly passed through a batch normalization layer and then through three blocks of a visual embedding extractor, three transformer encoders, a 3D CNN and a batch normalization layer. These blocks were followed by a 3D max pooling layer and three dense layers. Dropout was also added to avoid overfitting. Vision transformers worked by splitting a given input image into fixed size patches, linearly embedding them and then feeding them into a traditional transformer encoder [60]. A key difference between a “traditional” transformer and a ViT lies in the way they calculate attention. Transformers use attention to measure the relationship between pairs of input tokens. A token in a traditional transformer would be, e.g., a text string, while in our case it was a pixel. Moreover, instead of calculating embeddings directly from the raw source image, in our case we used feature maps to calculate them. Note that within each transformer, data are passing through a Batch Normalization Layer before being fed to the next block. This layer applies a transformation that maintains the mean close to 0 and the standard deviation close to 1.

#### 3.6.3. ResNets for Spectrograms

The third and final modality, i.e., the sound included in videos, is processed by a different deep neural network architecture which is based on convolutions, i.e., the ResNet50 [61], which is a residual network architecture comprising 50 layers; 48 layers are convolutional layers, accompanied by a max pooling layer and an average pooling layer. A typical ResNet architecture contains residual blocks and skip connections, which are implemented by adding the output of an earlier layer to the output of a later layer. This way, the information from an earlier layer is preserved and passed on to later layers, leading to the formation of better representations of the input data.

#### 3.6.4. Intermediate Fusion

A crucial part for any multimodal recognition pipeline is the approach used to fuse the available modalities. Early fusion is based on the combination of raw data representations into a single entity, prior to feature extraction. On the other hand, late fusion aggregates results upon classification per modality and may be more costly due to the need for separate training per modality [62]. However, in our work we chose to adopt an intermediate fusion approach, i.e., features extracted for each modality are combined before the classification process. Specifically, the first step of intermediate fusion is to extract separate features per modality through different ML learning streams. These features are then combined into a single feature vector representation, which is ultimately used for action classification. Features extracted from optical flow, RGB and audio modalities are fused using a weighted average layer and are then processed by several fully connected (dense) layers as per regular supervised learning methods. Note that weights in this layer are trainable; they are randomly initialized, taking values drawn from a uniform distribution and specifically in the range [0, 1) and during each training epoch they are refreshed through a softmax layer. The weighted averaging layer is followed by a Batch Normalization Layer (see Section 3.6.2).

**Figure 4:**
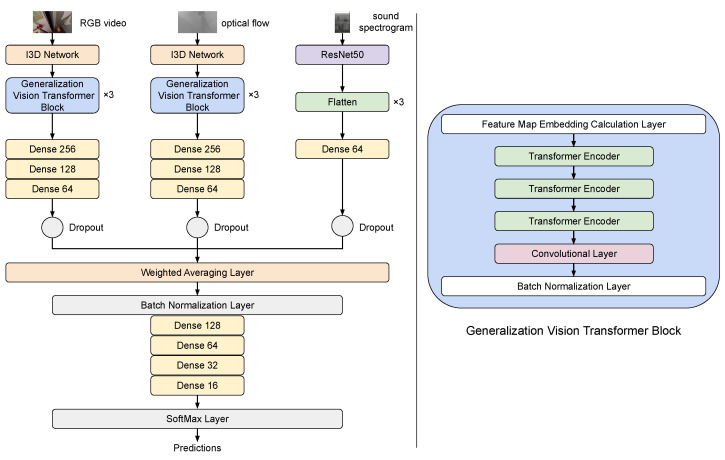
Network architecture of the proposed methodology.

In Figure 4 we illustrate in detail the proposed multi-modal action recognition model. Note that features from visual (video) streams were extracted using I3D, while three dense layers were required to efficiently learn feature representations, though the audio stream used spectrograms, i.e., 2D greyscale images as input, which are much simpler than the previously mentioned video streams. In that case, a single dense layer was adequate. Moreover, upon the fusion process, extracted features passed through a softmax-activated dense layer to perform action predictions.

### 3.7. Network Training

We trained our model for 1350 epochs when using a single modality and for 3000 epochs when using all modalities. In all cases we used the Adam optimizer with a learning rate of $5\times 10^{-5}$ and the categorical cross-entropy loss function. All hyperparameters were set using a grid search approach.

## 4. Experimental Results

In this section our goal is to present the dataset and the experimental protocol that we have used for the evaluation of this work, as well as comparisons to state-of-the-art research works and discussions of the results.

### 4.1. Dataset

We conducted our experiments using data from the Epic Kitchens 55 dataset [12]. As we have already mentioned, Epic-Kitchens is one of the largest egocentric vision video benchmark datasets. Its first version, namely Epic-Kitchens 55, was introduced in 2018 and offers a unique viewpoint on how people interact with objects in various kitchen environments. It included recordings from 32 participants in their own kitchen environments and was densely annotated with actions and object interactions. Activities depicted in this dataset were not scripted, as is evident by the way each recording starts. Each participant commenced recording upon entering their kitchen. Recordings took place in four countries (i.e., USA, Italy, UK and Canada) by actors of 10 different nationalities, leading to diverse kitchen habits, environments and cooking styles. The dataset features 55 h of video, consisting of 11.5 M frames, i.e., RGB images, which were densely labeled for a total of 39.6 K action segments. Additionally, since the dataset is also a benchmark for object recognition in video, it offers 452.3 K object bounding boxes.

What differentiates Epic Kitchens from other egocentric datasets is that its actors narrate their actions (i.e., using “free” language) after recording, helping the annotators to identify their true intentions. We should herein note that using free text descriptions in multiple languages to categorize actions performed in a video is not actually helpful for creating a ML model able to classify them. For this reason, the authors of the dataset grouped action classes with minimal semantic overlap to accommodate the classic approaches towards multi-class action recognition where each example belongs only to a single class. This led to the creation of a total of 125 “verb classes” $C_{V}$ and a total of 331 “noun classes” $C_{N}$. For example, the verb “take” groups words such as take, grab, pick, get, fetch and pick up and the noun “cupboard” groups words such as cupboard, cabinet, locker, flap, cabinet door, cupboard door and closet.

Furthermore, the Epic-Kitchens dataset characterizes kitchens as “seen” and “unseen” to assess generalizability in new environments. A seen kitchen training split resembles closely the classic supervised classification task (i.e., training/test data split). In this protocol a part of the given data is used for training (about 80% in most cases) and the rest for testing purposes (about 20%). In this case, both training and testing data will originate from the same kitchen and thus from a similar feature space. As stated by the dataset’s authors, Damen et al. [12], a given sequence is never split, i.e., it may only be part of either the training or the test set. An unseen kitchen training setup splits the participants in such a way that all video sequences of the same kitchen are either part of only the training or the test set. This last protocol may be beneficial to the evaluation of domain adaptation or generalization methods, since videos recorded in different kitchens will form different feature spaces, i.e., will belong to different domains. Thus, by considering a given kitchen as the source domain and another as the target domain, one may attempt to align the source feature space to the target one by using known methodologies.

For the experimental evaluation of our proposed methodology we followed the training/test setup that was introduced by Munro and Damen [48]. In this setup only the three largest kitchens are used, while participants P01, P22 and P08 are referred to as domains D1, D2 and D3, respectively. From the available data in these domains we evaluated the proposed methodology only using the eight largest action classes (verbs), namely “put”, “take”, “open”, “close”, “wash”, “cut”, “mix” and “pour”. These constitute 80% of the total action sequences in the aforementioned domains (kitchens). We should herein note that due to the public unavailability of the exact test data setup, we used the available test data from Epic-Kitchens 55 enhanced by the provided validation data from its updated version, namely Epic-Kitchens-100 [13] (the updated dataset) for the participants of interest. This lead to minor differences in test data, specifically between 1.6% and 5.3% per domain. Since our intention was to provide fair comparisons to the state-of-the-art works, we considered all unavailable data as “wrong predictions” of our model to prevent the aforementioned differences to act to the benefit of our approach. More details regarding the exact herein used training/test splits are depicted in Table 1.

### 4.2. Experimental Protocol and Results

We performed experiments using the setup of [48] and considered the following six cases of domain adaptation: D2→D1, D3→D1, D1→D2, D3→D2, D1→D3 and D2→D3. In each case, our model was trained using only data from the domain on the left side of the arrow (source domain), while it was evaluated using only data from the domain on the right side of the arrow (target domain). Moreover, we performed experiments per data modality. That is, apart from the proposed MDGAR method, we also evaluated its unimodal variation, namely UDGAR, wherein only one data modality among RGB, optical flow and audio was considered. In that case, only the respective part of the network was used. The metric used for evaluation was the Top 1–accuracy (i.e., the typical accuracy, comparing the model’s answer with the highest probability to the expected answer), averaged over 10 different model training sessions. To ensure robustness, only the last nine epochs of training were considered.

Since the herein proposed approach aims to produce a domain adaptable action recognition model, we compare our experimental results to the ones from similar recent domain adaptation research works in Table 2. Note that most of the methods we used for comparisons are based on unsupervised domain adaptation, i.e., no information from the target domain is considered. In our case though, we utilized the class distribution of the target domain to perform class balancing actions through data augmentation (see Section 3.5), making our model semi-supervised with regards to domain adaptation terminology. However, we assumed that both models may be used in similar real-life scenarios, e.g., such as recognizing human actions in previously unseen environments and this is the reason we proceeded with the aforementioned comparisons. It is noteworthy that we have followed a hybrid unseen kitchen testing protocol. Moreover, as in [48], in all experiments we reported on the averaged Top-1 target accuracy over the last 9 epochs of training.

Specifically, the first work we used for comparisons is the one of Munro et al. [48]. In this work the authors introduced a dual channel adversarial domain adaptation method (MM-SADA) based on two modalities, i.e., RGB and optical flow. Their method is considered unsupervised since the label space of the target domain is unknown. The addition of a self-supervision classifier also determines whether modalities are sampled from the same or a different action, leading to actively learning modality correspondence. In Table 2 we may observe that the proposed approach outperforms MM-SADA in most experimental settings, except for the case of D3→D2 using only the RGB modality. Moreover, we outperform the multi-modal source-only approach of Li et al. [51], namely AdaBN, the maximum mean discrepancy (MMD) presented by Long et al. [49], the maximum classifier discrepancy (MCD) proposed by Saito et al. [63], the TransVAE approach proposed by Wei et al. [52] and the CIA approach of Yang et al. [50], apart, again, from the D3→D2 using only the RGB modality. We should emphasize that we processed audio data differently from the CIA, i.e., we transformed them into spectrograms and then incorporated them in our learning pipeline.

From the above-mentioned experimental results of our approach and comparisons to other approaches, we observe that the use of audio data is able to provide a notable performance boost to our domain generalization method. In all unimodal test cases, audio-only experiments performed better compared to the other modalities. Also, in test cases D1→D3 and D3→D2, audio-only experiments performed better than their multi-modal counterparts, while in the rest they were able to achieve results close to the multi-modal test cases. One could argue that the audio modality is all we need to to build a robust model, while a more complex multi-modal model is not necessary. Although that may appear correct in our experiments, we should also consider a real-world scenario. In such a scenario, audio derived from daily activities may contain significant noise, and a costly data cleaning process would be necessary. For example, let us consider a user performing cooking tasks while listening to music; that would severely increase the complexity of the audio extraction and utilization task. On the other hand, visual data such as RGB and optical flow data are expected to be more “reliable”, without the need for a cleaning process.

## 5. Conclusions and Future Work

In this paper we proposed a multi-modal domain generalization method for building a robust human action recognition machine learning pipeline. To this end, we utilized data from three modalities, namely RGB, optical flow and audio. Visual data were similarly handled; both image representations underwent a sampling and scaling pre-processing step, while audio data were extracted from raw video sequences and were transformed into spectrograms. We introduced a data augmentation step which considered the target domain label distribution, i.e., it consisted of a semi-supervised method. The herein proposed deep learning architecture utilizes transfer learning by using pre-trained neural networks as a backbone. A complex three-stream architecture that is based on vision transformers and fully connected (dense) layers with intermediate fusion follows. Single-modality and multi-modality experiments were conducted using the well-known Epic-Kitchens-55 dataset in several cross-domain settings. We showcased how the proposed approach outperformed recent state-of-the-art domain adaptation methodologies, whilst producing results close to those produced by same-domain training procedures.

We should emphasize that the proposed approach holds all the advantages of egocentric vision, which typically allows for a more seamless interaction of users with the environment and is less obtrusive to stationary ones. Therefore, apart from raw video data, it also considers optical flow. The latter carries rich contextual information regarding the wearer’s gaze and movements. Moreover, it is independent of the wearable device used, as long as video data are provided. We also consider our approach to be domain-independent, as it does not rely on any domain-specific features; meanwhile, it may be trained and deployed in a scalable manner, since it may leverage techniques such as parallel processing and distributed computing due to its multi-stream architecture. This may enable our approach to handle increasingly large datasets to be expanded on with further data streams so as to provide more complex models and to efficiently handle the need for increasing computational resources, thus making it appropriate for a wide range of applications.

However, as with all egocentric approaches, the herein presented work has several limitations. Although, within the experiments presented in Section 4 and due to the dataset used, the field of view was adequate to capture all necessary information, this may not always be feasible. In another domain this limitation may lead to incomplete visual information, which in turn will ultimately lead to incomplete understanding. Of course, the same could happen in real-life scenarios, in cases of an “insufficient” point of view which could be attributed to the subjectivity of the human wearer of the video capturing device. Moreover, rapid or “shaky” motion of the wearer could cause motion artifacts, e.g., motion blur or image distortion. This degradation of captured visual data could significantly affect recognition.

Among future extensions of this work, we can list the following. Firstly, the intermediate fusion process that has been used herein may be replaced by a late fusion one. This can be achieved by using voting mechanisms, e.g., upon making action predictions with unimodal models, averaging them per class and choosing the max probability result as the final prediction. Moreover, since audio modality in most setups provided better accuracy over the visual modalities, weighted voting schemes could also be investigated. Also, other types of extracted features or representations of modalities could be investigated. Finally, the proposed architecture could be modified to utilize other types of pre-trained networks, or to use transfer learning from other similar visual datasets.

## Figures and Tables

**Table 1:** Training and test splits per domain.

Domain	D1	D2	D3
Kitchen	P08	P01	P22
Training Action Segments	1543	2495	3897
Test Action Segments [48]	435	750	974
Test Action Segments (ours)	412	713	990
Difference (%)	−5.3	−4.9	+1.6

**Table 2:** Results of the proposed approach compared to other state-of-the-art research works. Numbers denote Top-1 accuracy. Numbers in bold indicate best performance among all approaches, within the given domain adaptation scenario.

Methodogy	Domain Adaptation Scenario					
	D1→D2	D2→D1	D2→D3	D1→D3	D3→D1	D3→D2
**MM Source-only** [48]	42.0	42.5	46.5	41.2	44.3	56.3
**AdaBN** [51]	47.0	44.6	48.8	40.3	47.8	54.7
**MMD** [49]	46.6	43.1	48.5	39.2	48.3	55.2
**MCD** [63]	46.5	42.1	51.0	43.5	47.9	52.7
**MM-SADA** [48]	49.5	48.2	52.7	44.1	50.9	56.1
**TransVAE** [52]	50.5	50.3	58.6	50.3	48.0	58.0
**CIA** [50]	52.5	49.8	53.2	47.8	52.2	57.6
**UDGAR** (RGB)	54.6	76.7	69.6	60.1	**88.9**	48.4
**UDGAR** (Optical Flow)	64.2	66.7	69.9	59.0	81.7	80.1
**UDGAR** (Audio)	82.1	86.0	77.4	**86.8**	75.7	**81.1**
**MDGAR**	**84.6**	**88.0**	**85.2**	84.4	76.8	59.1

## Data Availability

Publicly available datasets were analyzed in this study. This data can be found here: https://github.com/epic-kitchens (accessed on 12 February 2024).

## Abbreviations

The following abbreviations are used in this manuscript:

CNN	Convolutional Neural Network
DFT	Discrete Fourier Transform
DL	Deep Learning
FPP	First Person Perspective
HAR	Human Activity Recognition
MDGAR	Multi-modal Domain Generalization model for Activity Recognition
ML	Machine Learning
ResNet	Residual Network
RGB	Red Green Blue
SAR	Supervised Activity Recognition
UDGAR	Unimodal Domain Generalization model for Activity Recognition
ViT	Vision Transformer
